# *Bartonella* DNA in Loggerhead Sea Turtles

**DOI:** 10.3201/eid1306.061551

**Published:** 2007-06

**Authors:** K. Hope Valentine, Craig A. Harms, Maria B. Cadenas, Adam J. Birkenheuer, Henry S. Marr, Joanne Braun-McNeill, Ricardo G. Maggi, Edward B. Breitschwerdt

**Affiliations:** * North Carolina State University College of Veterinary Medicine, Raleigh, North Carolina, USA; † Center for Marine Sciences and Technology, Morehead City, North Carolina, USA; ‡ National Marine Fisheries Service, Beaufort, North Carolina, USA

**Keywords:** Loggerhead sea turtle, Bartonella, ITS region, Pap 31, Caretta caretta, PCR analysis

**To the Editor:** *Bartonella* are fastidious, aerobic, gram-negative, facultative, intracellular bacteria that infect erythrocytes, erythroblasts, endothelial cells, monocytes, and dendritic cells, and are transmitted by arthropod vectors or by animal scratches or bites (*1*–*6*). Currently, 20 species or subspecies of *Bartonella* have been characterized, of which 8 are known zoonotic pathogens (*7*). *B. henselae* has been recently identified from canine blood (*8*) and from harbor porpoises (*9*). Pathogenic bacteria are an important threat in terrestrial and marine environments, and in the case of *B. henselae,* reservoir hosts may be more diverse than currently recognized.

The purpose of this study was to determine whether sea turtles are infected with *Bartonella* spp. Blood samples were obtained from 29 free-ranging and 8 captive, rehabilitating loggerhead sea turtles (*Caretta caretta*) from North Carolina coastal waters. Reptilian erythrocytes are nucleated, and commercial lysis methods clogged filtration columns because of the high DNA content of whole blood. Consequently, DNA was extracted from frozen whole blood by using a modified alkaline lysis method adapted from an avian cell culture DNA extraction method (*10*). PCR screening for *Bartonella* was performed by using primers for the 16S-23S internal transcribed spacer (ITS) region (Table). *Bartonella* ITS–positive samples were further screened by using primers for a consensus sequence of the phage-associated gene Pap31 (*9*). Primers for the 28S rRNA were used as a housekeeping gene. The PCR-positive control contained 0.002 pg/µL of *B. henselae* H1. Water was the negative PCR control. Amplicons of the expected sizes were consistently obtained from housekeeping gene and positive control reactions, while amplicons were never obtained from negative controls. ITS amplicons were obtained from 16 (43%) of 37 sea turtle blood samples tested, including samples from 13 free-ranging and 3 rehabilitated turtles. Pap31 PCR was performed for *Bartonella* ITS-PCR–positive samples. Pap31 amplicons were obtained from 5 samples of which 3 were successfully sequenced. Amplification and sequencing of the 16S-23S ITS region detected 2 *Bartonella* species: a *B. henselae*–like organisms and 1 more similar to *B. vinsonii* subsp. *berkhoffii.* The 3 Pap31 amplicons successfully sequenced confirmed *B. henselae* infection. Sequences obtained from 1 sample matched *B. henselae* strains H1-like, the *B. henselae* SA2-like strain, and *B. vinsonii* subsp. *berkhoffii* genotypes II and IV, which suggests that this turtle was co-infected with multiple *Bartonella* spp. and strains. Three other samples yielded amplicons 99%–100% identical with *B. henselae* strain SA2, and 3 yielded sequences most similar to *B. vinsonii* subspecies *berkhoffii* genotypes II and IV. Two samples contained an ITS region sequence most similar to *B. henselae* SA2, but with a 15-bp deletion beginning 617 bases downstream from the 16S rRNA gene. Whether these ITS sequence differences represent distinct strains or nonrandom translocation events is uncertain.

**Table:** Primers used for PCR amplification

Primer	Sequence
28s s	5′-AAACTCTGGTGGAGGTCCGT-3′
28s as	5′-CTTACCAAAAGTGGCCCACTA-3′
ITS 325s	5′-CTTCAGATGATGATCCCAAGCCTTTTGGG-3′
ITS 1100as	5′-GAACCGACGACCCCCTGCTTGCAAAGCA-3′
Pap 31 1s	5′-ACTTCTGTTATCGCTTTGATTTCRRCT-3′
Pap 31 688(as)	5′-CACCACCAGCAAAATAAGGCAT-3′

Four sea turtle blood samples contained partial ITS sequences most similar to *B. vinsonii* subsp. *berkhoffii*. However these amplicons were much shorter than expected for *B. vinsonii* subspecies *berkhoffii* genotype II and genotype IV sequences in GenBank. Because Pap31 gene amplification was unsuccessful for these samples, it is unclear whether small amplicons represent a species related to *B. vinsonii* subsp. *berkhoffii* or a new *Bartonella* sp.

To our knowledge, detection of *Bartonella* spp. DNA in sea turtle blood represents the first molecular evidence of *Bartonella* infection in nonmammalian vertebrates. *B. henselae* infection, now reported in porpoises and sea turtles, may represent an emerging infection of marine animals. According to previous studies, immune status appears to affect disease severity, variation in clinical manifestations, the pattern of histopathologic features, and the relative ease of diagnostic detection of the organism (*4*,*7*). Although healthy at the time of sample collection, the captive rehabilitated sea turtles were known to have been sick or injured before sampling, potentially reflecting immunocompromise. Whether detection of *Bartonella* spp. in blood of sea turtles is a function of prior immunosuppression induced by stressors is not known. Such stressors could include mechanical injury, malnutrition, environmental toxins, parasites, or concurrent bacterial or viral infections. Alternatively, sea turtles may be a natural marine reservoir for *B. henselae* or for a *Bartonella* sp. genetically related to *B. vinsonii* subsp. *berkhoffii.*

In summary, documentation of *B. henselae* and an organism genetically similar to *B. vinsonii* subsp. *berkhoffii* in the blood of loggerhead sea turtles provides evidence that this genus is not ecologically limited to terrestrial reservoirs. The geographic distribution, prevalence of infection, carrier potential, mode of transmission, and pathogenicity of bloodborne *Bartonella* spp. in sea turtles await additional studies.
